# T Cells in Osteoarthritis: Alterations and Beyond

**DOI:** 10.3389/fimmu.2017.00356

**Published:** 2017-03-30

**Authors:** Yu-sheng Li, Wei Luo, Shou-an Zhu, Guang-hua Lei

**Affiliations:** ^1^Department of Orthopaedics, Xiangya Hospital of Central South University, Changsha, China; ^2^Department of Orthopaedic Surgery, School of Medicine, Johns Hopkins University, Baltimore, MD, USA; ^3^Aging and Metabolism Research Program, Oklahoma Medical Research Foundation, Oklahoma City, OK, USA

**Keywords:** inflammation, inflammatory diseases, osteoarthritis, T cells, Th17 cells

## Abstract

Although osteoarthritis (OA) has been traditionally regarded as a non-inflammatory disease, reports increasingly suggest that it is inflammatory, at least in certain patients. OA patients often exhibit inflammatory infiltration of synovial membranes by macrophages, T cells, mast cells, B cells, plasma cells, natural killer cells, dendritic cells, granulocytes, etc. Although previous reviews have summarized the knowledge of inflammation in the pathogenesis of OA, as far as we know, no report review our current understanding about T cells, especially, each T cell subtype, in the biology of OA. This review highlights the current understanding of the role of T cells in the pathogenesis of OA, with attention to Th1 cells, Th2 cells, Th9 cells, Th17 cells, Th22 cells, regulatory T cells, follicular helper T cells, cytotoxic T cells, T memory cells, and even unconventional T cells (e.g., γδ T cells and cluster of differentiation 1 restricted T cells). The findings highlight the importance of T cells to the development and progression of OA and suggest new therapeutic approaches for OA patients based on the manipulation of T-cell responses.

## Introduction

Affecting approximately 3.8% (95% CI: 3.6–4.1) of the global population, osteoarthritis (OA) is regarded as a prevalent cause of morbidity and disability worldwide (1). OA shows many disease characteristics, such as cartilage degradation, moderate synovial inflammation, pain, alteration of bony structure, and impaired mobility (2). However, despite the severity of the disease, relatively little is known about its exact etiology. Recent compelling investigations have attributed the onset of OA to various person-level factors such as age, sex, obesity, and diet and joint-level factors such as injury, malalignment, and abnormal joint loading (3–5). Although more and more researchers have recently presented hypotheses concerning the involvement of these factors in OA, especially for person-level factors, few of their hypotheses have been demonstrated experimentally, and some have even been challenged by the latest observational studies and clinical trials (4, 6, 7).

Of the several factors potentially involved in the pathogenesis of OA, T cell-mediated immune responses and their influence on the biology of OA are the focus of this review (8–11). The scientific community once understood OA to be induced by mechanical stress in the form of cartilage destruction, with minimal if any involvement of immune responses. Thus, OA was regarded as a non-inflammatory disease, in contrast with rheumatoid arthritis (RA), an inflammatory disease (4, 7, 12, 13). However, recent studies suggest that at least in certain patients, OA is an inflammatory disease; patients have frequently been found to exhibit inflammatory infiltration of synovial membranes (9–11). Most recent studies have shown that the number of inflammatory cells in the synovial tissue is lower in patients with OA than in patients with RA, but higher than that in healthy subjects (14–18). Indeed, little difference has been found in the percentages of T cells, B cells, and natural killer cells in the peripheral blood between patients with OA and RA (19). Leheita et al. (19) reflected on the similarity of the immune cell profiles of RA and OA and suggested that abnormalities in T cells may also contribute to the pathogenesis of OA. Further experiments indicated that inflammation in OA is anatomically restricted and varies in intensity. The synovial membranes in regions rimming the cartilage of OA patients, which contain T cells bordered by B lymphocytes and plasma cells (20), showed a pronounced inflammatory response. In contrast, only a few infiltrating lymphocytes were observed in the synovial membranes taken from macroscopically non-inflamed areas in OA patients (20). This may explain the suggestion made by some researchers that immune responses are not involved in the pathogenesis of OA. When synovial samples from patients with knee OA were analyzed, the synovial lining cells showed strong immunoreactivity and phagocytic potential with cluster of differentiation (CD) 68 antibodies (8). These findings suggested that macrophages may be associated with the pathogenesis of knee OA. Of 20 osteoarthritic synovial membranes, 5 showed lymphoid follicles containing T cells, B cells, and macrophages, and 10 (including the latter five) displayed a diffuse cellular infiltrate containing T and B cells, macrophages, and granulocytes (21). These results suggested that B cells and granulocytes may also be involved in the pathogenesis of knee OA.

To date, various immune cells have been identified in the synovial membranes of OA patients, such as macrophages, T cells, mast cells, B cells, plasma cells, natural killer cells, dendritic cells, and granulocytes (8, 10, 22–27). For a detailed description of the infiltration of synovial tissues by immune cells, a recent review of this subject should be consulted (10). Of these inflammatory cells, macrophages and T cells most abundantly infiltrate the synovial tissues of OA patients. For example, macrophages represent approximately 65% of the immune cells that infiltrate the synovial tissues of patients with OA, and T cells make up 22% of the infiltrate (17). Although previous reviews have summarized the knowledge of inflammation in the pathogenesis of OA, as far as we know, no report reviews our current understanding about T cells, especially, each T cell subtype, in the biology of OA (9, 10, 28). More importantly, the scientific community has recently contributed to the growing literature on the involvement of T cells in the pathogenesis of OA with some interesting findings regarding the alteration of T cells during OA. Thus, this review focuses on our current understanding of the significance of T cells to OA biology.

## T Cells and OA

Analysis of enzyme-linked immunosorbent assay (ELISA) data has shown that compared with age-matched healthy controls, patients with OA show higher levels of the soluble form of CD4 (sCD4) in their serum. This suggests that peripheral T helper (Th) cells are involved in the pathogenesis of OA (29). Similarly, when stimulated with phorbol myristate acetate (PMA) and ionomycin, peripheral mononuclear cells from OA patients showed a higher expression of CD4 and CD8 markers than their counterparts from healthy controls (30). Indeed, the ratio of CD4^+^/CD8^+^ in the blood of OA patients is higher than that in the blood of healthy controls, although healthy controls and OA patients have fairly similar numbers of CD4^+^ and CD8^+^ T cells in their blood (31). Further evidence of the involvement of peripheral T cells in the pathogenesis of OA was provided by the discovery that the response to autologous chondrocytes of peripheral T cells isolated from OA patients is greater than of peripheral T cells isolated from controls and that this response is partially blocked by antibodies against human leukocyte antigen (HLA) classes I and II, CD4, and CD8 (32). Interestingly, T cells in a subset of OA patients were found to recognize the peptides representing amino acid regions 16–39 and 263–282 of human cartilage proteoglycan aggrecan (PG), and peripheral blood mononuclear cells from these PG-reactive OA patients showed an increased production of pro-inflammatory cytokines/chemokines in response to PG peptide stimulation (33). Based on these compelling findings, the autoimmune responses of peripheral T cells may aid understanding of immune-mediated mechanisms in OA.

Enzyme-linked immunosorbent assay analysis revealed higher levels of sCD4 not only in the peripheral blood but also in the synovial fluid of patients with OA, compared with age-matched healthy controls, which suggests that Th cells in the synovial fluid are involved in the pathogenesis of OA (29). When stimulated with PMA and ionomycin, mononuclear cells from the synovial fluid of OA patients showed a high expression of CD4 and CD8 markers (30). These compelling results suggested that T cells in the synovial fluid are associated with the pathogenesis of OA. This conclusion was supported by subsequent investigations. For example, the percentage of T cells in the synovial fluid of OA patients was found to be significantly higher than that in their peripheral blood (34), and T cells in the synovial fluid of OA patients expressed class II HLA (an indicator of activated T cells) (35). The percentages of CD4^+^ and CD8^+^ cells in the synovial fluid of OA patients were even similar to those found in RA patients (31).

T cells are the major constituents of synovial infiltrates in the membranes of OA patients, and both CD4^+^ T cells and CD8^+^ T cells have been found within synovial aggregates (35). For example, synovial tissue extracted from OA patients displayed perivascular CD3^+^ T cell infiltration at an early stage (36). Similarly, using immunohistochemical analysis, CD3^+^, CD4^+^, and CD8^+^ T cells were detected predominantly in the sublining layer and more limitedly in the deep layer of the synovium of patients with OA, whereas the presence of CD4^+^ T cells in the synovial sublining layer was detected more strongly in OA patients than in normal subjects (15). CD4^+^ T cells were found to be predominant among the T-cell infiltrates in the synovial tissue, and the number of CD4^+^ T cells was higher in the synovial sublining layer of patients with OA than in that of normal subjects. Indeed, the medial synovium of patients with knee OA has been shown to contain more CD4^+^ T cells than the lateral synovium (8). Interestingly, synovial aggregates from OA patients express CD80, an inducible costimulatory ligand involved in T-cell activation (35, 37), suggesting that synovial aggregates in OA patients are areas of antigen recognition and T-cell activation. Similarly, researchers investigating 30 patients with OA found CD3^+^ T cell aggregates in the synovial membrane in 65% of the patients, and the activation antigens CD69, CD25, CD38, CD43, CD45RO, and HLA class II were also found in the synovial membrane (38). In addition, HLA-antigen D-related (DR)-expressing T cells were found in the synovial membranes of OA patients using immunohistochemical analysis, although to a lesser degree than in RA patients (39). The conclusion that activated T cells are aggregated in the synovial membranes of OA patients was further supported by the discovery that virtually all T cells in OA joints express activation markers, such as HLA-DR and CD69 (40). Interestingly, OA patients older than 75 have higher percentages of CD3^+^, CD4^+^, and CD8^+^ cells in their synovial membranes than OA patients younger than 75 (41). This may suggest that age is among the risk factors for OA.

Collectively, significant abnormalities in the T-cell profile have been found in the peripheral blood, synovial fluid, and synovial membranes of OA patients. Based on these findings, T cells are assumed to be associated with the pathogenesis of OA.

### Th1 and OA

Under the stimulation of interleukin (IL)-12, naïve CD4^+^ T cells differentiate into Th1 cells, which produce IL-2, interferon (IFN)-γ, tumor necrosis factor (TNF)-α, lymphotoxins, and granulocyte-macrophage colony-stimulating factor (42–44). Most current evidence indicates that Th1 cells do not alter significantly on entering the peripheral blood of OA patients. For example, flow cytometry analysis has shown that there is little difference in the percentage of circulating Th1 cells (CD4^+^IFN-γ^+^ T cells) between OA patients and healthy controls (45). Similarly, no variation in either the percentage or the absolute number of circulating Th1 cells (CD4^+^IFN-γ^+^ T cells) has been found between patients with OA and healthy controls (46). However, in a study with 25 OA patients and 13 healthy controls, the number of circulating Th1 cells (IFN-γ^+^CD4^+^CD8^−^ T cells) and the level of serum IFN-γ were found to be significantly higher in patients with OA than in healthy controls (47). The difference in the markers (CD4^+^IFN-γ^+^ vs. IFN-γ^+^CD4^+^CD8^−^) used in the two studies to define Th1 cells may account for this discrepancy. Another explanation may lie in the variation between OA patients, such as differences between the stages of OA. The alteration of the Th1 cell profile in the peripheral blood of OA patients thus requires further investigation.

In contrast with the findings for peripheral blood, the synovial fluid of OA patients shows an increase in Th1 cells. Although early experiments suggested that the concentrations of IL-2, IFN-γ, and TNF-β in the synovial fluid of OA patients are below the limit of detection by ELISA analysis (48), reverse transcription polymerase chain reaction (RT-PCR) analysis has since revealed that cells from the synovial fluid of OA patients express IL-2 and IFN-γ when stimulated with PHA and ionomycin (35). Indeed, intracellular IFN-γ has been detected at higher levels in both CD4^+^ and CD8^+^ cells from the synovial fluid than in the peripheral blood of OA patients (30). In addition, high concentrations of IL-1β and TNF-α have been observed in the synovial fluid of patients with OA, whereas these markers are below the limit of detection in healthy subjects (31).

Th1 cells can also be found in the synovial membranes of OA patients. For example, IL-2, IFN-γ, and their receptors are usually detected in the synovial membranes of OA patients (38, 49). Similarly, INF-γ^+^ cells have been detected in the synovial membranes of patients with OA, predominantly in the sublining layer of the synovium, although to a lesser degree than in RA patients (15). In a mouse model of OA induced by anterior cruciate ligament transection (ACLT), the expression of IFN-γ increased during OA onset (30 days after ACLT) and then decreased at a later stage of OA (90 days after ACLT) (50). Most importantly, a well-designed study showed that Th1 cells are predominant in both OA and RA joints (40). Indeed, the number of IFN-γ^+^ cells in the synovium of patients with OA is approximately five times greater than that of IL-4^+^ cells (15).

In summary, although the profile of Th1 cells in the peripheral blood requires further analysis, Th1 cells have been shown to accumulate in the synovial fluid and synovial membranes of OA patients, which suggests that Th1 cells play important roles in the pathogenesis of OA. In addition, Th1 cell responses in the synovial fluid and synovial membranes of OA patients may be a marker of OA disease activity.

### Th2 and OA

When stimulated by IL-4, naïve CD4^+^ T cells differentiate into Th2 cells (44). Through the production of IL-4, IL-5, IL-10, and IL-13, Th2 cells affect the function of B cells, dendritic cells, eosinophils, etc. and play important roles in the host’s defense against multicellular parasites and in the pathogenesis of allergies (42, 43, 51–54). Most recent studies have shown that Th2 cells undergo limited alteration in the peripheral blood, synovial fluid, and synovial membranes of OA patients. For example, in a study of 18 OA patients, the IL-10 transcript was found in nearly all of the patients using competitive PCR analysis, whereas IL-4 and IL-5 were not detected in the synovial membranes of any of the patients (38). Similarly, the concentrations of IL-4 and IL-10 in the synovial fluid were below the limit of detection by ELISA analysis (48). Using flow cytometry analysis, low concentrations of Th2 cytokines such as IL-4 and IL-10 were detected in both the synovial fluid and the peripheral blood of OA patients (30). Although cells from the synovial fluid of OA patients stimulated with PHA and ionomycin expressed IL-10 at 48 h poststimulation, no signal for IL-4 was detected by RT-PCR analysis (35). The observed expression of IL-10 in OA patients’ synovial membranes or synovial fluid cells may come from other cells, such as regulatory T cells (Treg cells).

Together, although these compelling findings suggest that Th2 responses play only a limited role in the pathogenesis of OA, further strong evidence is needed to support this hypothesis.

### Th9 and OA

Th9 cells, recently defined as subsets of Th cells, preferentially produce IL-9 (44, 55–57). Th9 cells facilitate immune responses against melanoma and intestinal worms and are closely associated with the immunopathology of allergic and autoimmune responses, such as systemic lupus erythematosus (SLE), experimental autoimmune encephalitis, and systemic sclerosis (55–57).

Th9 cells are also involved in the pathogenesis of arthritis. For example, a high level of IL-9 has been detected in the peripheral blood and synovial fluid of patients with RA and patients with psoriatic arthritis (PsA), and the level of IL-9 in the synovial fluid is higher than that in the peripheral blood for RA and PsA patients (58). Similarly, activated CD3^+^ T cells from the peripheral blood and synovial fluid of patients with PsA or RA produce high levels of IL-9 (58). These results suggest that Th9 cells play critical roles in the pathogenesis of RA and PsA. Indeed, Th9 responses have also been observed in OA. For example, a high level of IL-9 has been detected in the peripheral blood and synovial fluid of OA patients, and the activation of purified CD3^+^ cells from the peripheral blood and synovial fluid of patients with OA produces a high level of IL-9, although lower than that observed in RA or PsA patients (58). Even more importantly, in a study with 25 OA patients and 13 healthy controls, the number of circulating Th9 cells and serum IL-9 level were found to be significantly higher in OA patients than in healthy controls (47). This study also found that the number of circulating Th9 cells was positively associated with the level of C-reactive protein in OA patients and that both the number of Th9 cells and the level of serum IL-9 were positively correlated with OA index (47).

In summary, these well-designed experiments lead to the conclusion that Th9 cells significantly shape the pathogenesis of OA, as well as that of RA and PsA; however, the Th9 response in the synovial membranes of OA patients needs further investigation. In addition, serum IL-9 or the number of circulating Th9 cells may be a marker of OA disease activity.

### Th17 and OA

Th17 cells secrete IL-17A (also known as IL-17), IL-17F, IL-21, and IL-22. Transform growth factor (TGF)-β, IL-6, IL-1β, and IL-23 have been reported to promote the differentiation of Th17 cells (44, 59–63). Th17 cells provide protection against bacterial infection and are associated with the development of autoimmune diseases *via* the recruitment of cells in the granulocyte lineage, especially neutrophils (64–67). Early investigations indicated that neither the percentages of circulating pure Th17 cells (CD4^+^IFN-γ^−^IL-22^−^IL-17^+^ T cells) and Th17 cells (CD4^+^IL-17^+^ T cells) nor the level of serum IL-17 differed significantly between OA patients and healthy controls (45). Similarly, no variation in the percentage or absolute number of circulating Th17 cells or the IL-17 plasma level was found between patients with OA and healthy controls (46). These findings indicated that little alteration occurs in the Th17 cell profile in the peripheral blood of OA patients. However, later observations suggested otherwise. In a rat model of OA induced by the injection of papain and l-cysteine into the right knee joint, the OA rats were found to have a higher serum IL-17 level than the control rats (68). In addition, in a study with 25 OA patients and 13 healthy controls, the number of circulating Th17 cells and the level of serum IL-17 were found to be significantly higher in patients with OA than in healthy controls (47). As in the case of Th1 cells, variation in the markers used to define Th17 cells (CD4^+^IL-17^+^ vs. IL-17^+^CD4^+^CD8^−^) and the patients selected for investigation (e.g., diagnosis standard, disease index, patients’ background) may account for this discrepancy. These controversial findings regarding Th17 cell profile in the peripheral blood of OA patients suggest that the roles of circulating Th17 cells in the pathogenesis of OA need further investigation. Nevertheless, it is widely accepted that Th17 cells are present in the synovial fluid and synovial membranes of OA patients. For example, in addition to the strong expression of IL-17 mRNA in the synovial membranes of OA patients (69), a high level of IL-17 has been measured in the synovial fluid of OA patients, whereas both are below the limit of detection in healthy subjects (31, 70). In addition, Th17 cells have been detected in the joints of OA patients, albeit in smaller numbers than in RA joints (40).

Collectively, these interesting results demonstrate the accumulation of Th17 cells in the synovial fluid and synovial tissue of OA patients; however, the exact role of Th17 cell response in the biology of OA needs further investigation.

### Th22 and OA

Originally, IL-22 was regarded as a product of Th17 cells; however, recent evidence has indicated that a distinct subset of human skin CD4^+^ T cells (Th22) produces IL-22 but not IL-17 or IFN-γ (71). Increasing evidence has been provided for the involvement of Th22 cells in the biology of RA. For example, the percentage of Th22 cells is higher in RA patients than in healthy controls, and the percentage of Th22 cells is positively correlated with IL-22 expression in RA patients (45). In addition, the percentage of Th22 cells is positively correlated with both C-reactive protein levels and joint disease activity scores in RA patients (45). These compelling discoveries indicate that Th22 response is associated with the pathogenesis of RA and that blocking IL-22 expression may be a reasonable therapeutic strategy for RA. Th22 cells are also involved in the biology of ankylosing spondylitis. Similar to the results for RA, the percentage and absolute number of circulating Th22 cells were found to be elevated in patients with ankylosing spondylitis compared with healthy controls (46). Similarly, ELISA analysis revealed that the level of IL-22 in the plasma was higher in patients with ankylosing spondylitis than in healthy controls (46). However, Th22 cells seem to play a limited role in the pathogenesis of OA. For example, compared with healthy controls, OA patients show no change in the percentage of circulating Th22 cells (CD4^+^IFN-γ^−^IL-17^−^IL-22^+^ T cells) and the level of IL-22 in the plasma (45). Similarly, another independent experiment revealed that neither the percentage nor the absolute number of circulating Th22 cells, nor the plasma level of IL-22, differ between patients with OA and healthy controls (46).

Collectively, unlike RA and ankylosing spondylitis, OA involves only a limited alteration of Th22 response in the peripheral blood; however, we lack data on the Th22 profile in the synovial fluid and synovial tissue of OA patients.

### Treg Cells and OA

Under the influence of TGF-β, naïve T cells differentiate into Treg cells, which produce IL-10 and TGF-β (43, 72–74). Treg cells are important immunoregulators in many inflammatory and autoimmune diseases, as they modulate the secretion of anti-inflammatory cytokines and the expression of receptors for cytokines (75). For example, RA patients have a lower percentage of Treg cells at sites of synovial inflammation and in the peripheral blood (76), which may induce the downregulation of T-cell tolerance and exacerbate the inflammatory process. Increasing evidence has been provided that the profile of Treg cells in the peripheral blood, synovial fluid, and synovial membranes of OA patients is similar to that of RA patients. For example, the percentage and absolute number of Treg cells (CD4^+^CD25^+/high^CD127^−/low^) in the peripheral blood, synovial fluid, and synovial membranes are similar in RA patients and OA patients, and Treg cells in both cases show greater accumulation in the synovial fluid and synovial membranes than in the peripheral blood (77). In addition, Treg cells in the peripheral blood, synovial fluid, and synovial membranes of both OA patients and RA patients display a memory phenotype (CD45RO^+^RA^−^) (77). Neither does the activation status (CD69 and CD62L) nor the expression of markers associated with Treg function (CD152, CD154, CD274, CD279, and GITR) in the peripheral blood, synovial fluid, or synovial membranes differ between OA patients and RA patients (77). Those compelling results indicate that as in the case of RA, a decrease in Treg-cell responses is involved in the pathogenesis of OA. Indeed, Ponchel et al. (11) analyzed blood from 121 healthy controls and 114 OA patients and found that the OA patients had fewer Treg cells than the healthy controls after adjusting for age (11). Although the frequency of CD4^+^CD25^+^Foxp3^+^ Treg cells has been found to be elevated in the blood of OA patients, OA patients show lower IL-10 secretion from Treg cells and fewer Tim-3^+^ Treg cells in the blood (78). Similarly, in a rat model of OA induced by the injection of papain and l-cysteine into the right knee joint, the percentage of CD4^+^CD25^+^Foxp3^+^ Treg cells in the peripheral blood was significantly lower in the OA rats than in the control rats (68).

In summary, a decrease in Treg-cell response may be involved in the pathogenesis of OA; however, the alteration of Treg-cell responses in the peripheral blood, synovial fluid, and synovial membranes of OA patients requires more comparative investigation with age-matched healthy controls.

### Follicular Helper T (Tfh) Cells and OA

Follicular helper T cells, located in the follicles of lymphoid tissue, induce B cells to produce immunoglobulins (79). Tfh cells express various distinguishing genes, such as CXCR5, PD-1, ICOS, CD40L, Bcl-6, and IL-21 (80). Increasing evidence has been provided for the influence of Tfh cells on the severity of autoimmune diseases, such as SLE and RA. For example, the number of circulating Tfh cells (CXCR5^+^ICOS^+^CD4^+^ cells or CXCR5^+^PD-1^+^CD4^+^ cells) has been shown to increase in a subset of SLE patients in line with the diversity and concentration of autoantibodies and SLE severity (81). Similarly, immunohistochemistry analysis has revealed specific staining for CD4, CXCR5, and ICOS on infiltrating immune cells in the synovial tissues of RA patients, and the presence of Tfh cells (CD4^+^CXCR5^+^ICOS^+^ T cells) in the synovial tissues of RA patients has been verified using both triple-fluorescence immunostaining and confocal laser scanning (82). This study provided evidence of the presence of Tfh cells in both SLE and RA patients, indicating the potentially important roles played by Tfh cells in the pathogenesis and progression of both diseases. However, the results of immunohistochemistry analysis, triple-fluorescence immunostaining, and confocal laser scanning revealed that Tfh cells are absent from the synovial tissues of OA patients (82). Yet, a recent investigation demonstrated the importance of Tfh cells to the pathogenesis and progression of OA. In the latter study, the frequency of ICOS^+^, PD-1^+^, and IL-21^+^ CXCR5^+^CD4^+^ T cells in the peripheral blood of 40 patients with OA and 13 healthy controls was examined by flow cytometry, and the concentration of serum IL–21 was also determined. Compared with the healthy controls, the OA patients showed higher percentages of CXCR5^+^CD4^+^, PD-1^+^CXCR5^+^CD4^+^, ICOS^+^CXCR5^+^CD4^+^, and IL-21^+^CXCR5^+^CD4^+^ T cells (83). Shan et al. (83) also found that OA patients exhibited higher levels of serum IL-21 than healthy controls and, even more importantly, that the expression of IL-21^+^Tfh cells in OA patients was positively correlated with the disease activity of OA (83). The latter study suggests that Tfh cells play a critical role in the pathogenesis and progression of OA. However, further well-designed research is needed to characterize Tfh cell profile in the peripheral blood, synovial fluid, and synovial membranes of OA patients.

### Cytotoxic T Cells and OA

The peripheral blood of OA patients has been analyzed using flow cytometry, revealing that patients with OA have significantly fewer CD8^+^ T cells and a higher CD4^+^:CD8^+^ ratio than healthy subjects (84). However, patients with OA have normal proportions of CD8^+^CD45RA^+^, CD8^+^CD29^+^, and CD8^+^S6F1^+^ cells in both their peripheral blood and their synovial fluid (85). These results indicate the alteration of peripheral CD8^+^ T cells in OA patients. Although CD8^+^ T cells can be found in the synovial membranes of OA patients, the major component of the T-cell infiltrate cannot. Most of the T cells found in the synovial membranes of patients with OA are helper T cells, whereas cytotoxic T cells occur sparsely in patients with OA (39, 86). Similarly, fewer CD8^+^ T cells than CD4^+^ T cells have been found in the lining, the sublining, and even the deep layer of the synovium of patients with OA (15). In addition, although both CD4^+^ and CD8^+^ T cells have been found in the synovial aggregates of OA patients, the aggregates contain a larger proportion of CD4^+^ T cells than of CD8^+^ T cells, and the CD8^+^ T cells are often located toward the periphery of the aggregates (35). CD8^+^ T cells play an important role in the pathogenesis of OA, although they are not the predominant T-cell type found in the synovial aggregates of OA patients. In mice with ACLT-induced OA, CD8^+^ T cells were activated once OA had been initiated, and the percentage of activated CD8^+^ T cells was significantly higher in the ACLT group than in the sham group during OA progression (87). In addition, the number of CD8^+^ T cells expressing tissue inhibitor of metalloproteinase-1 (TIMP-1) was found to be correlated with OA severity and inhibiting the expression of TIMP-1 in the joints retarded the progression of OA (87). Cartilage degeneration occurred more slowly in CD8^+^ T cell knockout mice than in wild-type mice (87).

In summary, a significant alteration to CD8^+^ T cells has been observed in the peripheral blood, the synovial fluid, and the synovial membranes, and CD8^+^ T cells have been found to significantly shape the pathogenesis of OA, although they do not play the most important role in the process.

### T Memory (Tm) Cells and OA

Once activated, most T cells undergo apoptosis; however, a minority persist as Tm cells. An increasing number of researchers have begun to investigate the profile of Tm cells in the pathogenesis of OA. For example, although healthy individuals showed no difference in the percentages of CD45RO^+^CD4^+^ T cells and CD45RA^+^CD4^+^ T cells in the peripheral blood, more CD45RO^+^ cells than CD45RA^+^ cells were found in the peripheral blood of patients with OA (88). In patients with OA, the majority of CD4^+^ T cells in the synovial fluid and synovial tissue are CD45RO^+^ and CD45RA^−^, suggesting that an accumulation of CD45RO^+^ memory CD4^+^ T cells is a generalized phenomenon in OA joints (88). Similarly, a study with 25 OA patients and 13 healthy controls revealed that the number of circulating CD4^+^CD45RO^+^ T cells was significantly higher in patients with OA than in healthy controls (47). Other evidence for the possible involvement of Tm cells in the pathogenesis of OA includes the detection of the regulated on activation, normal T cell expressed, and secreted chemokine (a potent chemoattractant for leukocytes, such as CD45RO^+^ memory T cells) and CD29 (a 1 integrin expressed by Tm cells) in the synovial fluid of OA patients (35, 37, 89).

In summary, CD45RO^+^ memory CD4^+^ T cells seem to be critical to the biology of OA, yet their exact roles in the pathogenesis of OA have yet to be determined.

### Unconventional T Cells and OA

Recent investigations have also highlighted the involvement of unconventional T cells in the pathogenesis of OA. For example, more and more evidence has been provided that γδ T cells are involved in the pathogenesis of RA. For example, the number of γδ T cells has been found to increase in the synovial membranes of RA patients (90–93), and γδ T cells in the synovial membranes have more and/or more avid Fc receptors for immunoglobulin G IgG in patients with RA compared with controls (90). Further research has shown that the majority of synovial γδ T cells in RA patients do not express Vγ9, Vδ2, or Vδ1-Jγδ1 (91). However, most recent studies have indicated that the number of γδ T cells in the synovial membranes of patients with OA does not increase (91–93). Immunohistochemical staining of synovial tissue with early-stage OA shows T-cell infiltration in the perivascular area, with the clonality of restricted T cell receptor usage in the V beta chain (36), which also indicates the minimal alteration of γδ T cells in OA patients. Recent studies have shown that the synovial membranes of OA patients express CD1 (94), which presents non-protein antigens to NKT cells, suggesting that CD1-restricted T cells may play a role in the pathogenesis of OA.

Overall, although numerous studies of the involvement of conventional T cells in OA have been conducted, it will be useful to determine the importance to OA of unconventional T cells such as CD1-restricted T cells, MR1-restricted mucosal-associated invariant T cells, major histocompatibility complex class Ib-reactive T cells, and γδ T cells (95).

## Conclusion

**Figure 1:**
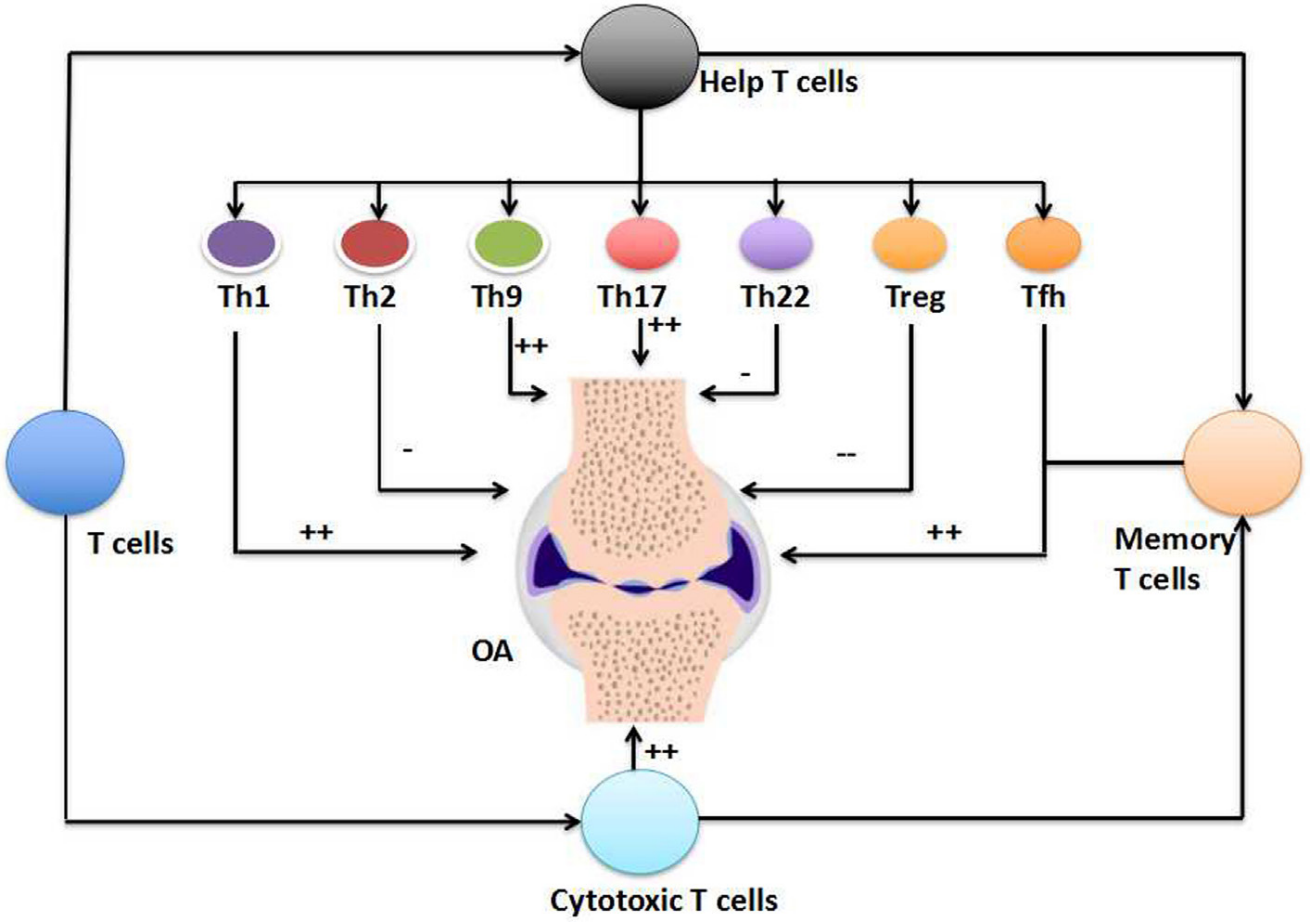
**The involvement of T cells in the pathogenesis of osteoarthritis (OA)**. T cells, including the T helper (Th) cells, cytotoxic T cells, and T memory (Tm) cells, have critical importance in the pathogenesis of OA (++). The involvement of unconventional T cells in the pathogenesis of OA is not shown here. Within T helper (Th) cells, Th1 cells, Th9 cells, Th17 cells, and follicular helper T (Tfh) cells increase in the peripheral blood, synovial fluid, or synovial membranes of OA patients (++). The numbers of cytotoxic T cells and Tm cells also increase in the OA. However, the numbers of Th2 cells and Th22 cells show limited alteration in the pathogenesis of OA (−), but the number of Treg cells decrease during the OA (−−).

Various risk factors for OA have been proposed, ranging from person-level factors such as age, sex, and obesity, to joint-level factors such as injury, malalignment, and abnormal loading of joints (3–5). Increasing evidence has also been provided that inflammation is associated with the development and progression of OA, at least in certain patients. OA patients often exhibit the infiltration of synovial membranes by inflammatory cells such as macrophages, T cells, mast cells, B cells, plasma cells, natural killer cells, dendritic cells, and granulocytes (8–11). Several scholars have investigated the alteration of T cells during the pathogenesis of OA, with reference to Th1 cells, Th2 cells, Th9 cells, Th17 cells, Th22 cells, Treg cells, Tfh cells, cytotoxic T cells, Tm cells, and even unconventional T cells (e.g., γδ T cells and CD1-restricted T cells). To date, it has been widely accepted that a significant alteration occurs in the profiles of Th1 cells, Th9 cells, Th17 cells, Treg cells, cytotoxic T cells, and Tm cells in the peripheral blood, synovial fluid, and synovial membranes of OA patients (Figure 1; Table 1). However, the involvement of Th2 cells, Th22 cells, Tfh cells, and unconventional T cells in the pathogenesis of OA needs further investigation (Figure 1; Table 1). In addition, the causal relationship between the alteration of T-cell responses and the development and progression of OA has yet to be identified. Various factors such as extracellular stimulation (e.g., antigens), intracellular signaling [e.g., mTOR complex 1 (mTORC1)], and cell metabolism (e.g., amino acid metabolism) are significant determinants of the fate of T cells (54, 66, 96–100). Therefore, it will be interesting to manipulate these factors to determine their precise effects on the development and progression of OA. For example, mTORC1 is known to regulate Th17 differentiation and IL-17 expression through several pathways, including STAT3, HIF-1α, S6K1, and S6K2, which raises the possibility of regulating the pathogenesis of OA through mTORC1 signaling (66). Indeed, the great importance of mTORC1 signaling to the pathogenesis of OA has recently been highlighted (101). The metabolism and transportation of amino acids also have a remarkable influence on T-cell activation and differentiation, especially for Th1 and Th17 cells, suggesting that amino acid metabolism also affects the pathogenesis of OA. Indeed, the establishment and development of OA are associated with alterations in the metabolism and profile of amino acids such as those of the glutamate and arginine families, as well as their related metabolites (e.g., creatinine, hydroxyproline, γ-aminobutyrate, dimethylarginines, and homoarginine) (4). Furthermore, intestinal microbiota have vital roles in T-cell responses, especially those of Th17 cells (102, 103), indicating the critical functions of intestinal microbiota in the pathogenesis of OA. Indeed, intestinal microbiota have been proposed as a risk factor in the development and progression of OA (7, 104). Understanding the significance of the altered T-cell profile to the pathogenesis of OA will open up novel directions for preventing and treating OA by modulating T-cell responses.

**Table 1:** **The alteration of Th subset cells in OA**.

T cells	Cytokines produced by T cells	Alterations in the OA		Reference
Th1 cells	IL-2, IFN-γ, TNF-α	Peripheral blood	No	(45, 46)
		Synovial fluid	Increase	(30, 31, 35)
		Synovial membrane	Increase	(15, 38, 40, 49, 50)
Th2 cells	IL-4, IL-5, IL-10, IL-13	Peripheral blood	No	(30)
		Synovial fluid	No	(30, 35, 48)
		Synovial membrane	No	(38)
Th9 cells	IL-9	Peripheral blood	Increase	(47, 58)
		Synovial fluid	Increase	(58)
		Synovial membrane	–	
Th17 cells	IL-17A, IL-17F, IL-21, IL-22	Peripheral blood	No	(45, 46)
			Increase	(47, 68)
		Synovial fluid	Increase	(31, 70)
		Synovial membrane	Increase	(40, 69)
Th22 cells	IL-22	Peripheral blood	No	(45, 46)
		Synovial fluid	–	
		Synovial membrane	–	
Treg cells	IL-10, TGF-β	Peripheral blood	Decrease	(11, 68, 78)
		Synovial fluid	–	
		Synovial membrane	–	
Follicular Helper T cells	CXCR5, PD-1, CD40L, Bcl-6, IL-21	Peripheral blood	Increase	(83)
		Synovial fluid	–	
		Synovial membrane	No	(82)
Cytotoxic T cells		Peripheral blood	Decrease	(84)
			No^a^	(85)
		Synovial fluid	No^a^	(85)
		Synovial membrane	Increase	(15, 35, 39, 86)

*IL, interleukin; IFN, interferon; TNF, tumor necrosis factor; TGF, transform growth factor; CXCR5, C-X-C chemokine receptor type 5; PD-1, programmed death 1; –, further investigations are needed; OA, osteoarthritis; Th, T helper; Treg, regulatory T*.

*^a^CD8^+^CD45RA^+^, CD8^+^CD29^+^, and CD8^+^S6F1^+^ cells*.

## Author Contributions

Y-sL and G-hL conceived this study. Y-sL wrote the manuscript. WL and S-aZ provided critical discussion in manuscript preparation. G-hL revised the manuscript.

## Conflict of Interest Statement

The authors declare that the research was conducted in the absence of any commercial or financial relationships that could be construed as a potential conflict of interest.
